# 176. Evaluation of the Predictive Value of Methicillin-Resistant *Staphylococcus aureus* (MRSA) Nares Polymerase Chain Reaction (PCR) Screening within Hospitalized Patients

**DOI:** 10.1093/ofid/ofac492.254

**Published:** 2022-12-15

**Authors:** Nicole Alilaen, Catherine Vu, Victoria Burke, Lillian Bellfi

**Affiliations:** University Medical Center New Orleans, Houston, Texas; University Medical Center New Orleans, Houston, Texas; LSU Health New Orleans School of Medicine, New Orleans, Louisiana; LSU Health New Orleans School of Medicine, New Orleans, Louisiana

## Abstract

**Background:**

While MRSA nares PCR screening utilization has established evidence for de-escalation of anti-MRSA agents in pneumonia, its utility in other sites of infection is not well studied. The purpose of this study was to evaluate the utility of MRSA nares PCR screening within a wide range clinical specimens and its impact on antimicrobial stewardship.

**Methods:**

This retrospective study was conducted at a 446-bed academic medical center. Patients ≥ 18 years old with an in-house MRSA nares PCR ordered between 1 November 2021 and 1 February 2022 were included in the primary analysis. Negative predictive value (NPV), positive predictive value (PPV), sensitivity, and specificity were calculated using cultures obtained within 7 days of nares screening. Vancomycin duration, de-escalation, and incidence of acute kidney injury were collected pre- and post- implementation of the in-house MRSA PCR screening. The pre-intervention cohort observed patients admitted from 1 November 2020 to 1 February 2021 screened by send out MRSA nares PCR.

**Results:**

At baseline, patients were a mean age of 55 years old, 55% black, and required ICU care in 50% of cases. A total of 665 cultures from 308 patients were included in the primary analysis (131 respiratory, 282 blood, 154 urine, 61 wound, 22 sterile fluid, 15 bone, and 12 spinal fluid specimens). The overall NPV of the MRSA nares PCR screening was 99.8% and the PPV was 21.1%. The sensitivity and specificity were 96.8% and 81.67%, respectively. Duration of vancomycin was a median of 3 days in both the pre- and post- intervention cohorts. However, rates of vancomycin de-escalation increased at day 3 from 31.5% to 48.1% and at day 5 from 69.9% to 80.0% post implementation on an in-house MRSA PCR screening. Incidence of acute kidney injury decreased from 45.2% to 21.6%.

**Conclusion:**

Based on its high NPV among cultures of different sites, MRSA nares PCR screening may be utilized to de-escalate anti-MRSA agents within tertiary care facilities.

**Disclosures:**

**All Authors**: No reported disclosures.

